# Self-reported health and the well-being paradox among community-dwelling older adults: a cross-sectional study using baseline data from the Canadian Longitudinal Study on Aging (CLSA)

**DOI:** 10.1186/s12877-022-02807-z

**Published:** 2022-02-10

**Authors:** Carly Whitmore, Maureen Markle-Reid, Carrie McAiney, Jenny Ploeg, Lauren E. Griffith, Susan P. Phillips, Andrew Wister, Kathryn Fisher

**Affiliations:** 1 School of Nursing, McMaster University, 1280 Main Street W, Hamilton, Ontario L8S 4K1 Canada; 2 School of Public Health Sciences, University of Waterloo & Schlegel-University of Waterloo Research Institute for Aging, 200 University Ave W, Waterloo, Ontario N2L 3G1 Canada; 3 Department of Health Research Methods, Evidence, and Impact, McMaster University, 1280 Main Street W, Hamilton, Ontario L8S 4K1 Canada; 4 School of Medicine, Queen’s University, 220 Bagot St, Kingston, Ontario K7L 5E9 Canada; 5 Department of Gerontology, Simon Fraser University, 515 W Hastings St, Vancouver, British Columbia V6B 5K3 Canada

**Keywords:** Self-rated health, Multimorbidity, Chronic disease, Older adult, Community, CLSA

## Abstract

**Background:**

Self-reported health is a widely used epidemiologic measure, however, the factors that predict self-reported health among community-dwelling older adults (≥65 years), especially those with multimorbidity (≥2 chronic conditions), are poorly understood. Further, it is not known why some older adults self-report their health positively despite the presence of high levels of multimorbidity, a phenomenon known as the well-being paradox. The objectives of this study were to: 1) examine the factors that moderate or mediate the relationship between multimorbidity and self-reported health; 2) identify the factors that predict high self-reported health; and 3) determine whether these same factors predict high self-reported health among those with high levels of multimorbidity to better understand the well-being paradox.

**Methods:**

A cross-sectional analysis of baseline data from the Canadian Longitudinal Study on Aging was completed (*n* = 21,503). Bivariate stratified analyses were used to explore whether each factor moderated or mediated the relationship between multimorbidity and self-reported health. Logistic regression was used to determine the factors that predict high self-reported health in the general population of community-dwelling older adults and those displaying the well-being paradox.

**Results:**

None of the factors explored in this study moderated or mediated the relationship between multimorbidity and self-reported health, yet all were independently associated with self-reported health. The ‘top five’ factors predicting high self-reported health in the general older adult population were: lower level of multimorbidity (odds ratio [OR] 0.75, 95% confidence interval [CI] 0.74-0.76), female sex (OR 0.62, CI 0.57-0.68), higher Life Space Index score (OR 1.01, CI 1.01-1.01), higher functional resilience (OR 1.16, CI 1.14-1.19), and higher psychological resilience (OR 1.26, CI 1.23-1.29). These same ‘top five’ factors predicted high self-reported health among the subset of this population with the well-being paradox.

**Conclusions:**

The factors that predict high self-reported health in the general population of older adults are the same for the subset of this population with the well-being paradox. A number of these factors are potentially modifiable and can be the target of future interventions to improve the self-reported health of this population.

**Supplementary Information:**

The online version contains supplementary material available at 10.1186/s12877-022-02807-z.

## Background

While definitions of multimorbidity vary in number (e.g., 2 or more versus 3 or more chronic conditions) and in the chronic conditions considered [1], there is consistent and strong evidence in the literature that an increasing level of multimorbidity is associated with lower self-reported health [2–6]. Self-reported health is a commonly used and reliable measure in health research because of its demonstrated association with morbidity and mortality [7, 8]. Since the 1950s, hundreds of studies have demonstrated that lower self-reported health is associated with higher levels of both morbidity and mortality – especially among older adults [9]. Self-reported health captures individual subjective assessments of health [10] by asking one simple question, “In general, would you say that your health is excellent, very good, good, fair, or poor?”. The response to this question is known to be influenced by knowledge of one’s own health, social norms, or expectations of illness, as well as illness acceptance [11–14].

As adults age, the likelihood of developing chronic conditions such as cardiovascular disease, arthritis, and diabetes increases [15, 16]. Multimorbidity (> 2 chronic conditions) is highly prevalent among older adults and is associated with decreased health-related quality of life, increased use of medical and social services, and increased risk for adverse events [15, 17]. Increasing longevity and an associated increase in multimorbidity among older adults has resulted in a change in the way that successful aging has been conceptualized. Traditionally, successful aging measures revolve around the absence of disease, the presence of physical and cognitive capacity, and ongoing social engagement [18]. More recently research emphasis has shifted from objective to subjective indices of health, including those that consider the presence of positive emotions such as happiness or satisfaction in aging – despite the presence of multimorbidity [19]. This is due, in part, to a subset of the older adult population, who despite having poorer health according to objective indicators, report positive levels of subjective health (e.g., self-reported health) [18]. This phenomenon is known as the well-being paradox and may be indicative of ‘multimorbidity resilience’ (i.e., resilience in responding to and coping with multimorbidity) [20]. Multimorbidity resilience is shaped by coping strategies and previous life experiences acquired throughout the lifecourse and related to health and illness at the individual, social, and environmental level [20].

Numerous studies have identified factors other than multimorbidity that are associated with self-reported health [5, 21] including demographic (e.g., sex), health-related (e.g., performance of activities of daily living or fewer depressive symptoms), and behavioural (e.g., greater social participation) factors. However, little is known about how these factors shape self-reported health or whether the relationship between multimorbidity and self-reported health changes in the presence of these other factors. This study was designed to address these gaps by exploring the interaction of these factors with multimorbidity in predicting self-reported health and accordingly creating a model to predict high self-reported health among community-dwelling older adults and the subset of this population with the well-being paradox.

### Purpose

The objectives of this study were to: 1) examine whether sociodemographic, health-related, or resilience factors moderate or mediate the relationship between multimorbidity and self-reported health; 2) identify the factors that predict self-reported health, and; 3) determine whether these same factors predict high self-reported health in those with high levels of multimorbidity to better understand the well-being paradox.

## Methods

A detailed study protocol, including the methods and measures used, has been published elsewhere [22]. Therefore, we only briefly summarize these below.

### Data source

A cross-sectional analysis of baseline data from the Canadian Longitudinal Study on Aging was completed. The CLSA is a national population-based study that follows 51,338 community-dwelling individuals recruited at baseline aged 45 to 85 years for a 20-year duration [23]. Interviews were conducted in English and French. Participants were excluded from CLSA if they resided in one of Canada’s three territories, lived on a federal First Nations reserve, were full-time members of the Canadian Armed Forces, lived in an institutional setting, or had a cognitive impairment precluding them from providing informed consent or providing data on their own at the time of recruitment [23]. The overall participation rate for CLSA was approximately 45% and the response rate was 10% [24].

The CLSA includes two cohorts: a tracking cohort and a comprehensive cohort. The tracking cohort includes a stratified random sample of 21,241 individuals from 10 Canadian provinces who provide data via telephone interview. The comprehensive cohort includes a stratified random sample of 30,097 individuals from the geographical area surrounding 11 data collection sites who provide questionnaire data via an in-home interview and take part in a physical assessment at a CLSA data collection site [24]. Full details of the CLSA are described in the published protocol [23].

### Sample

**Fig. 1:**
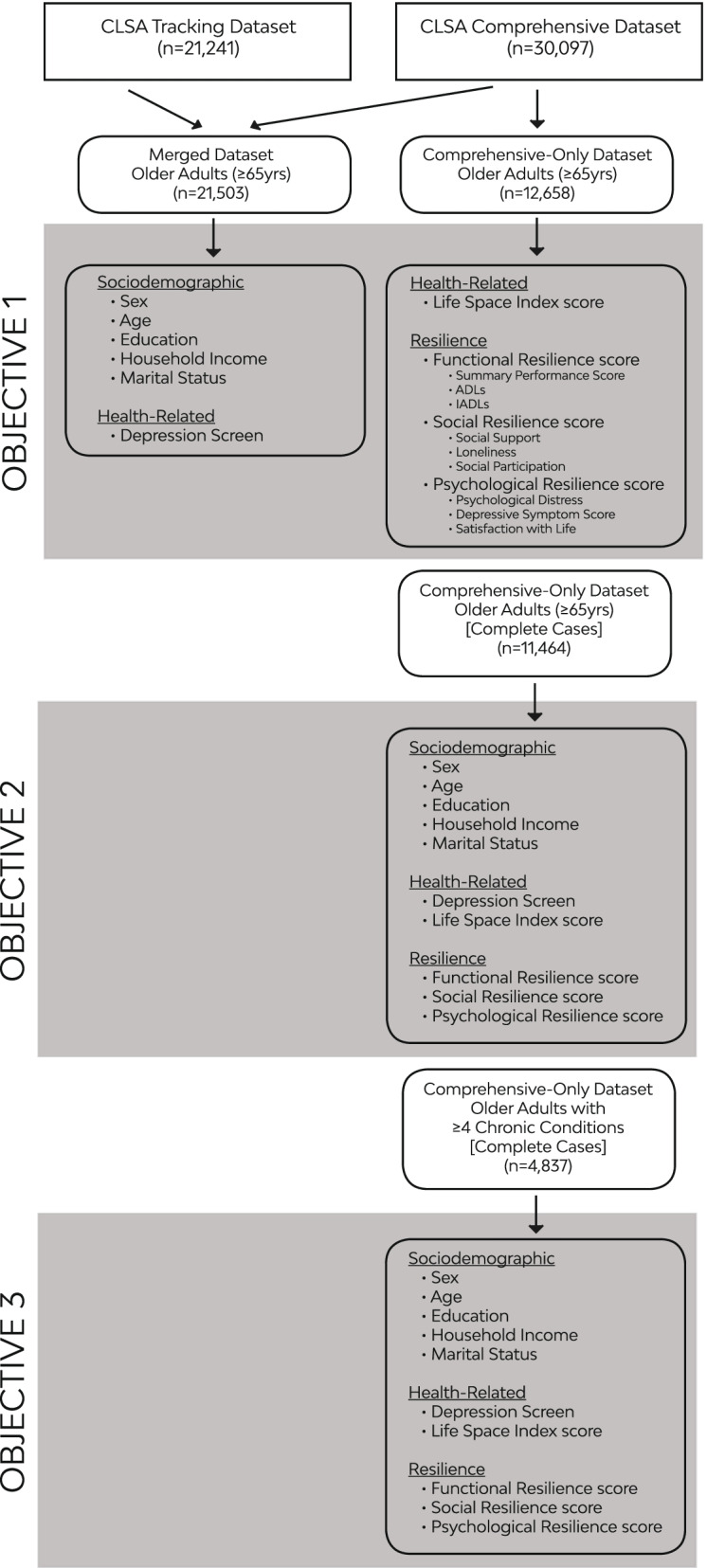
Data sources for each study objective

A subset of the full CLSA sample was used for these analyses. All participants 65 years of age and older from both the CLSA baseline tracking (version 3.4) and comprehensive (version 4.0) (*n* = 21,503) datasets were included in the analysis. Due to limitations on variables available (i.e., those variables that required in-person data collection), for some analyses, only the comprehensive participants (*n* = 12,658) were utilized. Data sources from the CLSA datasets for each of the study objectives are displayed in Fig. 1.

### Measures

#### Self-reported health

Self-reported health in the CLSA is evaluated as a five-item question, with respondents reporting their health as 1 = excellent, 2 = very good, 3 = good, 4 = fair, or 5 = poor. In addition to this ordinal scale, self-reported health was further dichotomized as either high self-reported health (responses of excellent and very good) or low self-reported health (responses of good, fair, and poor).

#### Level of multimorbidity

The level of multimorbidity was defined in this study as the number of chronic conditions and based on a list of 20 common chronic conditions [25], 18 of which were available in the CLSA. These included: the presence of hypertension, mood disorder (anxiety or depression), chronic musculoskeletal conditions, arthritis (rheumatoid or osteoarthritis), osteoporosis, respiratory conditions (asthma or chronic obstructive pulmonary disease), cardiovascular disease (angina, myocardial infarction, or peripheral vascular disease), heart failure, stroke, stomach conditions (ulcer), colon conditions, diabetes, thyroid disorder, cancer (did not include non-melanoma skin cancer), kidney disease, chronic urinary conditions, dementia, and obesity. By measuring the level of multimorbidity based on the number of chronic conditions, gradient effects could be explored.

#### Well-being paradox

Older adults were classified as having the well-being paradox if they reported high self-reported health (excellent or very good) and a high level of multimorbidity (four or more chronic conditions). Four or more chronic conditions was selected based on a clinical understanding of the burden of these conditions. This is because while some of the conditions could be described as risk factors (e.g., hypertension, obesity) or symptoms (e.g., incontinence, colon disorder) [26], those older adults with four or more chronic conditions are likely to experience greater challenges with their health than those with fewer than four conditions.

#### Sociodemographic and health-related

Independent sociodemographic variables identified from the literature [21] and available in CLSA included: sex (female or male), age (continuous variable and 65-69, 70-74, 75-79, 80+ years), marital status (single, married, or widowed, divorced, or separated), education (≤ secondary school, degree or diploma, or greater than a degree or diploma), household income (<$20,000, $20,000-49,999, $50,000-99,000, $100,000-149,999, ≥$150,000), and current dwelling type (house, apartment/condominium, or retirement home, assisted living).

In addition to sociodemographic factors, health-related factors were examined and included a depressive symptom score, a depression screen, as well as a Life Space Index score. Depressive symptom score was obtained from the Centre for Epidemiologic Studies Depression Scale 10-item (CES-D-10) and analyzed as both continuous (i.e., reflective of the severity of depressive symptoms) and categorical (i.e., reflective of the presence of depression) [27]. The CES-D-10 contains ten questions related to feelings of depression, loneliness, hopefulness, and other related physical symptoms such as decreased sleep [27] and provides a measure of the severity of depressive symptoms. For each question, participants respond with either “all of the time”, “occasionally”, “some of the time”, and “rarely or never”. Total scores range from 0 to 30, with higher scores indicating higher levels of depressive symptoms. This score was also used to screen for the presence of depressive symptoms (≥10/30) [27]. CES-D-10 scores were included in the demographic and bivariate stratified analyses but were excluded from the logistic regression analyses because the multimorbidity resilience measures used in the regressions included this measure as described below.

Life-space mobility was measured using the Life Space Index [28], which was available within the CLSA comprehensive dataset only. This is a self-report of the frequency and extent of movement within and from one’s home to the neighbourhood and beyond [28]. The Life Space Index reports mobility across different locations, such as rooms in the house, yard, neighbourhood, alternative neighbourhoods, and outside of one’s city/town, frequency of going from place to place, and whether assistance was needed [28]. Scores are calculated for each level of mobility with a maximum score of 120 (e.g., going out of town without assistance) [28, 29].

#### Multimorbidity resilience

Multimorbidity resilience was measured using a resilience index developed by Wister and colleagues [30] using CLSA data. This index maps functional, social, and psychological factors to multimorbidity resilience with a composite score of the three subdomains, each of which is comprised of three index measures representing adversity and adaptation [30].

Functional resilience was measured using the Older Americans Resources and Services (OARS) Activity of Daily Living (ADL) Scale, the OARS Instrumental Activities of Daily Living (IADL) Scale, as well as the Summary Performance Score [31]. The OARS ADL Scale consists of 7 indicators of daily tasks such as eating and personal hygiene. Each of the 7 tasks is scored on a scale from 0 (i.e., completely unable) to 2 (i.e., completely able). Total scores range from 0 to 14, with higher scores indicating higher functional status [31]. The OARS IADL Scale, also a measure of functional ability, consists of 7 measures of instrumental activities such as taking medication and preparing meals [31]. The Summary Performance Score was calculated from individual scores of a standing balance measure, a walk time measure, and a timed chair raise measure. For each of these, a score from 1 to 4 based on statistical quartiles was assigned. If participants did not complete a task, they were assigned a 0. The overall Summary Performance Score ranged from 0 to 12 with a higher score reflecting greater physical ability [31].

Social resilience was measured using the Medical Outcomes Study (MOS) Social Support Survey, a social participation variable, and a perceived loneliness measure. The MOS Social Support Survey is a 19-item tool that measures emotional or informational support, affection support, tangible support, and positive social interaction [32]. For each of the questions, a score of 1 (“none of the time”) to 5 (“all of the time”) is assigned. Total scores range from 19 to 95, with higher scores indicating higher levels of social support [32]. Social participation is a measure developed by the research team at CLSA which asks participants to report how often they engaged in activities with friends or family over the past 12 months. Possible responses to this measure are “once a day”, “at least once a week”, “at least once a month”, “at least once a year”, to “never” [23]. Lastly, using the CES-D-10, perceived loneliness is measured with responses of “all of the time”, “occasionally”, “some of the time”, and “rarely or never” [27].

Psychological resilience was measured using the CES-D-10, the Kessler Psychological Distress K10 Scale, and the Diener Satisfaction with Life Scale. The Kessler Psychological Distress Scale is a 10-item scale that measure global distress including symptoms of anxiety [33]. Answers to these questions can range from 0 (“never”) to 3 (“most of the time”) with a total score of 30 representing the greatest distress [33]. The Diener Satisfaction with Life Scale involves 5 items that assess global satisfaction with responses ranging from 1 (“strongly disagree”) to 7 (“strongly agree”) [34]. Total scale scores range from 5 to 35, with higher score indicating higher levels of life satisfaction [34].

A total resilience score, consisting of the functional, social, and psychological sub-domain scores, provides a total scale score capturing multimorbidity resilience. Calculation of the functional, social, and psychological composite scores, along with the total resilience score, is described elsewhere [30]. The resilience variables were available for comprehensive participants only as some of the component variables were not collected in the tracking cohort. In addition to the scores for each subdomain, as well as the total resilience score, individual measures within each of the sub-domains (e.g., Satisfaction with Life, social participation, ADLs) were analyzed to explore how these items shape self-reported health. Full descriptions of the CLSA dataset and variables are available in the CLSA cohort profile [24] and in the paper by Wister and colleagues [30].

### Statistical analysis

To examine the relationship between multimorbidity and self-reported health and whether the factors moderated or mediated this relationship, bivariate stratified analyses were completed. Analyses began with examining the relationship between the level of multimorbidity and self-reported health. Stratified analyses were then performed to explore whether each factor (e.g., demographic, health, or resilience factors) modified or mediated the relationship between the level of multimorbidity and self-reported health. These analyses were guided by work on effect modification, interaction, and mediation by Corraini and colleagues [35] as well as Frazier and colleagues [36]. Two-way analysis of variance (ANOVA) were used to determine the statistical significance of the associations factor-by-factor. Two-way ANOVA models included the level of multimorbidity, the additional independent variable, and the interaction of the two in predicting self-reported health. Where statistical significance of a relationship was noted, visual inspection of the interaction plots was completed to assess whether meaningful interactions were present due to the large sample size.

For a comprehensive understanding of the factors that predict high self-reported health, a multiple, complete case logistic regression was used. The regression model included sociodemographic, health-related, and resilience factors that were independently and individually significantly associated with self-reported health. This was preceded by tests of multicollinearity to confirm that factors included in the models were not highly correlated with one another.

Tests of model fit were completed including Cragg Uhler’s R^*2*^ [37] and Wald test [38]. In addition, a variable importance function was used to estimate the contribution of each independent variable in the model using the absolute value of the *t*-statistic for the model parameter. The same analytical methods described above were used to determine the predictors of high self-reported health among the subset of individuals within this population with high levels of multimorbidity (i.e., 4+ chronic conditions). Regression analyses used only the CLSA comprehensive dataset (*n* = 12,658) because some of the factors (life space index and resilience index scores) are only available in this dataset. Due to the large sample size available for these analyses and the potential for statistically significant but not clinically significant findings, the relative effect size was calculated using Cohen’s d (where *d* = [LogOddsRatio x (√3/π)]). Findings are reported using Cohen’s classification criteria to determine small (*d*= > 0.2), moderate (*d*= > 0.5), or large (*d*= > 0.8) effect sizes (Cohen, 1988). Analyses were completed using SAS (version 3.8) and R (version 4.0.2).

## Results

### Sociodemographic and health-related characteristics

Of the 21,503 community-dwelling older adults (≥65 years) included in this study sample, 50% were female, 55% were between the ages of 65 and 74, 62% were married or in a common-law relationship, and 97% were white. Even though the older adults in these analyses reported an average of 3.25 chronic conditions, 58% of the sample rated their general health as high (very good or excellent). The most common chronic conditions were hypertension (51%), arthritis (39%), chronic musculoskeletal conditions (27%), diabetes (22%), and cardiovascular disease (20%). In addition, 15% of the study sample screened positive for depressive symptoms on the CES-D-10. Key demographic and health-related data are included in Table 1.

**Table 1:** Key demographic and health factors

Characteristic	Total	
	Combined Dataset (*n* = 21,503) or Comprehensive Dataset only (*n* = 12,658)*	
**Sex**	*n*	Proportion
Female	10,749	50.02%
Male	10,742	49.98%
Total	21,491	
**Age**		
Mean Age (SD)	73.36 (5.82)	
Median Age (Range)	73 (65 – 89)	
**Marital / Partner Status**	*n*	Proportion
Single / Always lived alone	1207	5.62%
Married / Common-law	13,287	61.83%
Widowed, Divorced / Separated	6993	32.54%
Refused	4	0.02
Total	21,491	
**Race (not mutually exclusive)**	*n*	Proportion
White	20,832	96.88%
Other Race	816	3.79%
Refused / No answer / Don’t know	26	0.12%
**Education**	*n*	Proportion
≤ Secondary School Graduation	13,186	61.35%
University Degree or College Diploma	5575	25.94%
> Degree / Diploma	2682	12.48%
Refused / Don’t Know	50	0.17%
Total	21,493	
**Economic Status (Household Income)**	*n*	Proportion
< $20,000	1536	7.15%
$20,000 - $49,999	7440	34.62%
$50,000 – $99,999	7386	34.36%
$100,000 - $149,999	2148	9.99%
≥ $150,000	1064	4.95%
Refused / Don’t Know	1917	8.92%
Total	21,493	
**Current Dwelling**	*n*	Proportion
House	15,899	73.98%
Apartment / Condo	5058	23.54%
Retirement Home / Assisted Living, Rooming / Lodging House, Other	365	1.69%
Total	21,322	
**Chronic Conditions**	*n*	Proportion
Hypertension	10,885	50.94%
Arthritis or Rheumatoid Arthritis	8212	38.92%
Chronic Musculoskeletal Condition	5853	27.34%
Obesity	5516	25.65%
Diabetes	4637	21.64%
Cardiovascular Disease	4288	20.15%
Heart Failure	3910	18.31%
Thyroid Disorder	3742	17.72%
Asthma or Chronic Obstructive Pulmonary Disease	3542	16.58%
Cancer	3550	16.57%
Depression or Anxiety	3360	15.69%
Osteoporosis	3115	14.63%
Urinary Incontinence	2621	12.24%
Stomach Ulcer	1985	9.28%
Irritable Bowel Disease	2123	9.92%
Stroke or Transient Ischemic Attack	1696	7.92%
Kidney Disease	876	4.10%
Dementia or Alzheimer’s disease	78	0.36%
**Level of Multimorbidity**		
Mean Number of Chronic Conditions (SD)	3.25 (2.13)	
Median Number of Chronic Conditions (Range)	3 (0 – 15)	
**Depressive Symptoms (CES-D-10)**		
Mean Score (SD)	5.16 (4.40)	
Median Score (Range)	4 (0 – 30)	
Life Space Index (Mobility)*		
Mean Score (SD)	80.50 (18.41)	
Median Score (Range)	82.00 (0 – 120)	
**Self-Rated (General) Health**	*n*	Proportion
Excellent	4022	18.71%
Very Good	8445	39.30%
Good	6526	30.37%
Fair	2031	9.45%
Poor	438	2.04%
Did not complete	29	
Total	21,491	
Functional Resilience*		
Mean Score (SD)	7.52 (2.45)	
Median Score (Range)	7.76 (0 – 10)	
Psychological Resilience*		
Mean Score (SD)	2.32 (1.77)	
Median Score (Range)	2.23 (0 – 6.67)	
Social Resilience*		
Mean Score (SD)	7.19 (1.87)	
Median Score (Range)	7.23 (0 – 10)	
Total Resilience*		
Mean Score (SD)	5.91 (1.13)	
Median Score (Range)	6.02 (0 – 8.89)	

Note: * marks those data only analyzed from the CLSA comprehensive dataset

### Objective 1: factors that moderate or mediate the relationship between level of multimorbidity and self-reported health among community-dwelling older adults

Findings indicated that as the level of multimorbidity increased, self-reported health decreased. One-way ANOVA results showed that self-reported health was significantly different across levels of multimorbidity (*F*(6) = 751.44, *p* = <.0001). Kruskal-Wallis results were consistent.

#### Sociodemographic and health-related factors

The main effects for all socio-demographic, health, and resilience factors were significant (*p* = <.0001) in the models (see Table 2). Significant interaction effects were found between multimorbidity age group (*F*(6,3) = 2.41, *p* = .0007); education (*F*(6,2) = 4.43, *p* = <.0001); life space index (*F*(6,3) = 2.22, *p* = .0022), and self-reported health. Despite the statistical significance of these interactions, visual examination of the interaction plots did not suggest a meaningful interaction (see Supplementary File 1).

**Table 2:** Two-Way ANOVA main effects and interaction effects

Combined Datasets (***n*** = 21,503) or Comprehensive Dataset only (***n*** = 12,658)*				
Factor	***DF***	Mean Square	***F***	Pr > ***F***
**Sex**				
Main effect	1	44.29	58.49	<.0001
Interaction effect	6	0.62	0.82	0.55
**Age Group**				
Main effect	3	7.69	10.16	<.0001
Interaction effect	18	1.82	2.41	0.0007
**Education**				
Main effect	2	71.96	96.02	<.0001
Interaction effect	12	3.32	4.43	<.0001
**Household Income**				
Main effect	4	51.36	69.16	<.0001
Interaction effect	24	0.46	0.62	0.92
**Marital Status**				
Main effect	4	3.41	4.49	<.0001
Interaction effect	24	0.38	0.48	0.98
**Depression Screen**				
Main effect	1	376.37	514.58	<.0001
Interaction effect	6	0.57	0.77	0.59
Life Space Index*				
Main effect	3	53.81	76.46	<.0001
Interaction effect	18	1.56	2.22	0.0022
Functional Resilience*				
Main effect	3	97.51	141.81	<.0001
Interaction effect	18	1.13	1.65	0.0411
Summary Performance Score*				
Main effect	3	97.38	140.14	<.0001
Interaction effect	18	0.99	1.42	0.11
Activities of Daily Living*				
Main effect	1	66.57	93.33	<.0001
Interaction effect	6	1.04	1.46	0.18
Instrumental Activities of Daily Living*				
Main effect	1	151.98	216.99	<.0001
Interaction effect	6	2.55	3.65	0.0013
Social Resilience*				
Main effect	3	44.51	62.63	<.0001
Interaction effect	18	0.69	0.98	0.48
Social Support*				
Main effect	3	22.96	32.14	<.0001
Interaction effect	18	0.49	0.68	0.83
Loneliness*				
Main effect	4	25.58	36.02	<.0001
Interaction effect	22	0.59	0.83	0.68
Social Participation*				
Main effect	5	21.63	30.39	<.0001
Interaction effect	29	0.86	1.21	0.20
Psychological Resilience*				
Main effect	3	142.08	208.47	<.0001
Interaction effect	18	0.85	1.25	0.21
Psychological Distress*				
Main effect	3	100.31	149.55	<.0001
Interaction effect	18	0.64	0.95	0.51
Depressive Symptom Score*				
Main effect	3	116.34	169.87	<.0001
Interaction effect	18	0.85	1.25	0.21
Satisfaction with Life*				
Main effect	3	163.96	244.94	<.0001
Interaction effect	18	1.15	1.72	0.0289
Total Resilience*				
Main effect	3	178.29	267.69	<.0001
Interaction effect	18	0.97	1.46	0.09

Note: * marks those data only analyzed from the CLSA comprehensive dataset

#### Multimorbidity resilience factors

Independent effects for each of the factors that comprise the functional, social, and psychological resilience scores as well as the scores themselves were identified (see Table 2). Further to these independent effects, significant interactions were found between multimorbidity, functional resilience score (*F*(6,3) = 1.65, *p* = .04); IADLs (*F*(6,1) = 3.65, *p* = .0013); satisfaction with life (*F*(6,3) = 1.72, *p* = .0289), and self-reported health. However, visual examination of the interaction plots did not suggest a meaningful interaction between these factors.

### Objective 2: factors that predict high self-reported health among community-dwelling older adults

All factors, except household income and marital status, were significantly associated with high self-reported health (see Table 3). Using Cohen’s classification criteria, female sex (d = − 0.26) and education level greater than a diploma or a degree (d = 0.21) had the largest effect sizes, although these would be classified as ‘small’ using Cohen’s thresholds. The effect sizes for the other statistically significant factors were even smaller.

**Table 3:** Logistic regression model for higher self-reported health – factors and effect

Comprehensive Dataset, Complete Cases Only (***n*** = 11,464) Higher Self-Reported Health (***n*** = 6915); Lower Self-Reported Health (***n*** = 4549)				
Factors	Notes	Pr > |z|	(OR) Point Estimate [Confidence Interval]	***d***
Intercept		3.12e-16	0.05 [0.02-0.10]	−1.652
Number of Chronic Conditions	Continuous variable	< 2e-16	0.75 [0.74-0.76]	−0.1573
Age	Continuous variable	1.18e-07	1.02 [1.01-1.03]	0.0118
Sex^a^	Categorical variable Male (2) vs. Female (1)	< 2e-16	0.62 [0.57-0.68]	−0.2627
Level of Education^a^	Categorical variable Degree/Diploma (2) vs. ≤ High School (1)	0.002702	1.16 [1.05-1.27]	0.0808
	> Degree/Diploma (3) vs. ≤ High School (1)	8.16e-10	1.46 [1.29-1.65]	0.2095
Household Income	Categorical variable $20,000 - 49,999 (2) vs. <$20,000 (1)	0.040579	1.20 [1.01-1.43]	0.1011
	$50,000 – 99,999 (3) vs. <$20,000 (1)	0.019588	1.24 [1.04-1.49]	0.1198
	$100,000 – 149,999 (4) vs. <$20,000 (1)	0.003941	1.36 [1.10-1.68]	0.1709
	≥$150,000 (5) vs. <$20,000 (1)	0.073544	1.25 [0.98-1.59]	0.1213
Marital Status	Categorical variable Married/Common-law (2) vs. Single/always lived alone (1)	0.177297	0.88 [0.73-1.06]	−0.0716
	Widowed/Divorced/Separated (3) vs. Single/always lived alone (1)	0.583730	0.95 [0.78-1.15]	−0.0294
Life Space Index Score	Continuous variable	9.09e-16	1.01 [1.01-1.01]	0.0057
Functional Resilience Score	Continuous variable	< 2e-16	1.16 [1.14-1.19]	0.0823
Social Resilience Score	Continuous variable	0.000181	1.05 [1.02-1.07]	0.0259
Psychological Resilience Score	Continuous variable	< 2e-16	1.26 [1.23-1.29]	0.129

Notes: CI 95%, two-sided alpha, rounded to 2 decimal places

^a^Where d notes small effect size

### Objective 3: factors that predict high self-reported health among the subset of community-dwelling older adults with high multimorbidity

Using the CLSA comprehensive dataset, 18.1% (*n* = 2296) of older adults with high multimorbidity had high self-reported health (i.e., the well-being paradox). In comparison, 24.2% (*n* = 3067) of these same older adults with high multimorbidity had low self-reported health.

#### Characteristics of older adults with the well-being paradox

Older adults in the well-being paradox group had higher education (*X* ^2^(2) = 42.48, *p* = <.0001) and household income (*X* ^2^(5) = 14.98, *p* = 0.0204), reported fewer depressive symptoms (*t*(5320) = 16.75, *p* = <.0001), had a higher Life Space Index score (*t*(5352) = − 14.54, *p* = <.0001), higher overall levels of resilience (*t*(5361) = − 21.30, *p* = <.0001), as well as higher levels of functional, social, and psychological resilience, compared to the ‘non-well-being paradox’ group – defined as those with low self-reported health and high multimorbidity (see Table 4). In addition, those in the well-being paradox group (i.e., high self-reported health and high level of multimorbidity) had a lower mean number of chronic conditions compared to the non-well-being group (5.02 vs. 5.63).

**Table 4:** Differences in characteristics for those with the well-being paradox

Characteristic	Comprehensive Dataset (***n*** = 12,658) Older Adults with High Multimorbidity (4+) (***n*** = 5363)				
	High Self-Reported Health (*n* = 2296)		Low Self-Reported Health (*n* = 3067)		
**Sex**	*n*	Proportion	*n*	Proportion	*p*
Female	1303	56.75%	1660	54.12%	0.0556
Male	993	43.25%	1407	45.88%	
**Age Group**					*p*
Mean Age (SD)	73.81 (5.57)		73.76 (5.77)		0.7754
Median Age (Range)	74 (65 – 86)		74 (65 – 86)		
**Marital / Partner Status**	*n*	Proportion	*n*	Proportion	*p*
Single / Always lived alone	134	5.84%	179	5.84%	0.9044
Married / Common-law	1401	61.07%	1853	60.50%	
Widowed, Divorced, Separated	759	33.09%	1031	33.66%	
**Education**^**b**^	n	Proportion	n	Proportion	*p*
≤ High School	711	30.97%	1164	37.95%	<.0001
Diploma or Degree	1114	48.52%	1445	47.11%	
> Degree/Diploma	471	20.51%	458	14.93%	
**Economic Status**^**a**^**(Household Income)**	*n*	Proportion	*n*	Proportion	*p*
< $20,000	132	5.75%	244	7.96%	0.0204
$20,000 - $49,999	717	31.26%	982	32.05%	
$50,000 – $99,999	833	36.31%	1068	34.86%	
$100,000 - $149,999	279	12.16%	313	10.22%	
≥ $150,000	115	5.01%	164	5.35%	
**Level of Multimorbidity**					*p*
Mean Number (SD)^b^	5.02 (1.26)		5.63 (1.66)		<.0001
Median Number (Range)	5 (4 – 11)		5 (4 – 14)		
**Depressive Symptoms (CES-D-10)**^**b**^					*p*
Mean Score (SD)	4.94 (4.14)		7.12 (5.08)		<.0001
Median Score (Range)	4 (0 – 26)		6 (0 – 28)		
**Life Space Index**^**b**^					*p*
Mean Score (SD)	81.43 (17.57)		73.73 (20.31)		<.0001
Median Score (Range)	82 (9 – 120)		74 (6 – 120)		
**Functional Resilience**^**b**^					*p*
Mean Score (SD)	7.49 (2.32)		6.16 (2.87)		<.0001
Median Score (Range)	6.67 (0 – 10)		6.67 (0 – 10)		
**Psychological Resilience**^**b**^					*p*
Mean Score (SD)	2.62 (1.59)		1.87 (1.62)		<.0001
Median Score (Range)	2.23 (0 – 5.57)		1.1 (0 – 5.57)		
**Social Resilience**^**b**^					*p*
Mean Score (SD)	6.43 (1.77)		5.92 (1.94)		<.0001
Median Score (Range)	6.53 (0 – 9.17)		6.10 (0 – 9.17)		
**Total Resilience**^**b**^					*p*
Mean Score (SD)	6.57 (2.45)		5.08 (2.59)		<.0001
Median Score (Range)	6.67 (0 – 10)		5.53 (0 – 10)		

^a^Statistically significant mean differences at <.05

^b^ < .0001 (chi-squared; independent t-tests)

#### Factors that predict high self-reported health among the subset of participants with high multimorbidity

With the exception of social resilience, the factors predicting high self-reported health among the general population of community-dwelling older adults were the same as those predicting high self-reported health among older adults with high multimorbidity (see Table 5). Using Cohen’s classification criteria, male compared to female sex (*d* = − 0.28) and an education beyond a bachelor’s degree compared to high school graduate or less (*d* = 0.22) were found to have small effects on higher self-reported health among this subset of the sample while the remaining factors had even less impact.

**Table 5:** Logistic regression model for presence of well-being paradox – factors and effect

Comprehensive Dataset: Older Adults with ≥ 4 Chronic Conditions, Complete Cases Only (***n*** = 4837) Well-Being Paradox (***n*** = 2074); Not Well-Being Paradox (***n*** = 2763)				
Factors	Notes	Pr > |z|	(OR) Point Estimate [Confidence Interval]	***d***
Intercept		< 2e-16	0.01 [0.00-0.03]	−2.5131
Number of Chronic Conditions (4 or more)	Continuous variable	7.30e-15	0.83 [0.79-0.87]	−0.1008
Age	Continuous variable	2.07e-09	1.04 [1.02-1.05]	0.0194
Sex^a^	Categorical variable Male (2) vs. Female (1)	1.26e-14	0.59 [0.53-0.68]	−0.2819
Level of Education^a^	Categorical variable Degree/Diploma (2) vs. ≤ High School (1)	0.00999	1.19 [1.04-1.38]	0.1
	> Degree/Diploma (3) vs. ≤ High School (1)	1.20e-05	1.50 [1.25-1.79]	0.2237
Household Income	Categorical variable $20,000 - 49,999 (2) vs. <$20,000 (1)	0.15808	1.20 [0.93-1.55]	0.1014
	$50,000 – 99,999 (3) vs. <$20,000 (1)	0.07632	1.27 [0.97-1.66]	0.1319
	$100,000 – 149,999 (4) vs. <$20,000 (1)	0.00747	1.52 [1.11-2.07]	0.1912
	≥$150,000 (5) vs. <$20,000 (1)	0.34790	1.19 [0.83-1.71]	0.0954
Marital Status	Categorical variable Married/Common-law (2) vs. Single/always lived alone (1)	0.11535	0.80 [0.61-1.06]	−0.1219
	Widowed/Divorced/Separated (3) vs. Single/always lived alone (1)	0.28599	0.86 [0.65-1.14]	−0.0833
Life Space Index Score	Continuous variable	6.13e-11	1.01 [1.01-1.02]	0.0067
Functional Resilience Score	Continuous variable	< 2e-16	1.16 [1.13-1.19]	0.0819
Social Resilience Score	Continuous variable	0.05424	1.04 [0.99-1.07]	0.0197
Psychological Resilience Score	Continuous variable	< 2e-16	1.24 [1.19-1.29]	0.1187

Notes: CI 95%, two-sided alpha, rounded to 2 decimal places

^a^Where d notes small effect size

Goodness-of-fit diagnostics were completed for both models (see Table 6). Findings from Cragg Uhler’s *R*^*2*^ test highlight that both models are relatively weak. In examining the Wald test and the variable importance analysis, the similarities between the two models were apparent – i.e., level of multimorbidity, sex, Life Space Index score, functional resilience score, and psychological resilience score were the ‘top five’ predictors of higher self-reported health in both models.

**Table 6:** Goodness-of-fit diagnostics for logistic regression models

Diagnostic Test of Model Fit	Higher Self-Reported Health among all Older Adults (***n*** = 11,464)	Higher Self-Reported Health among Subset with Well-Being Paradox (***n*** = 4837)
**Cragg Uhler’s** ***R***^***2***^	0.25	0.18
**Wald Test** (***p***)		
Number of Chronic Conditions	< 2.22e-16	8.8692e-15
Age	1.201e-7	2.2227e-9
Sex	< 2.22e-16	1.5198e-14
Level of Education	6.6387e-9	5.6963e-5
Household Income	0.077106	0.08751
Marital Status	0.20682	0.24972
Life Space Index Score	9.9874e-16	6.763e-11
Functional Resilience Score	< 2.22e-16	< 2.22e-16
Social Resilience Score	0.00018238	0.054303
Psychological Resilience Score	< 2.22e-16	< 2.22e-16
**Variable Importance** (***t***)		
Number of Chronic Conditions	25.18	7.78
Age	5.29	5.99
Sex	10.64	7.71
Level of Education		
Degree/Diploma (2) vs. ≤ High School (1)	2.99	2.58
> Degree/Diploma (3) vs. ≤ High School (1)	6.14	4.38
Household Income		
$20,000 - 49,999 (2) vs. <$20,000 (1)	2.05	1.41
$50,000 – 99,999 (3) vs. <$20,000 (1)	2.33	1.77
$100,000 – 149,999 (4) vs. <$20,000 (1)	2.88	2.67
≥ $150,000 (5) vs. <$20,000 (1)	1.79	0.94
Marital Status		
Married/Common-law (2) vs. Single/always lived alone (1)	1.35	1.57
Widowed/Divorced/Separated (3) vs. Single/always lived alone (1)	0.55	1.07
Life Space Index Score	8.04	6.54
Functional Resilience Score	14.17	10.31
Social Resilience Score	3.74	1.92
Psychological Resilience Score	16.46	10.43

## Discussion

Study objectives were to: 1) examine whether sociodemographic, health-related, or resilience factors moderate or mediate the relationship between multimorbidity and self-reported health; 2) identify the factors that predict self-reported health, and; 3) determine whether these same factors predict high self-reported health in those with high levels of multimorbidity to better understand the well-being paradox. This study has generated several key findings.

None of the sociodemographic, health-related, and resilience factors moderated or mediated the relationship between multimorbidity and self-reported health, yet all were independently associated with self-reported health. This confirms existing evidence that has demonstrated the breadth of factors that shape how older adults perceive their health [21]. However, to our knowledge, this is the first study to explore the factors that potentially moderate or mediate the relationship between multimorbidity and self-reported health among a general population of community-dwelling older adults. These findings highlight that the burden of multimorbidity is not only a strong factor associated with self-reported health, but that the association between multimorbidity and self-reported health is seemingly not influenced by other demographic, health-related, and resilience factors.

Our work has uniquely identified five key factors that predict high self-reported health among a general population of community-dwelling older adults, as well as a subset of this population with high multimorbidity (i.e., the well-being paradox). These factors included a lower level of multimorbidity, female sex, higher Life Space Index score, and higher levels of functional and psychological resilience. While other studies have identified factors predictive of high self-reported health, including female sex [39] and physical performance (e.g., balance, chair stand test) [40, 41], this is the first study to identify that the factors that predict high self-reported health among a general population of older adults is the same for the subset of the population with high multimorbidity. This finding is a unique contribution to the literature because while the well-being paradox is commonly acknowledged and identified, it is poorly described and understood. This may be because of the limited linkage between the well-being paradox as a concept and its relevance to clinical practice.

Occurring alongside increasing longevity and multimorbidity, the contradictory nature of reporting positive perceptions of health despite living with multiple chronic conditions challenges the way that health care professionals measure wellness in older age [18, 19]. Evidence has shown that primary care providers often rate patient’s health differently than they rate it themselves [42]. This incongruence between providers’ and older adults’ perceptions of health is due to the fact that physicians tend to evaluate health based solely on the presence of disease, while older adults are more likely to evaluate their health based on other factors, including their illnesses, whether or not they are feeling well [42] and the presence of happiness [43]. One interpretation of these findings is that this difference in emphasis and perceptions on multimorbidity between providers and individuals may contribute to the presence of the well-being paradox, not some innate difference in the older adults themselves. From a practice, research, and policy perspective, these findings support the growing shift toward person-centred care that emphasizes the importance of assessing individual perceptions of health.

### Implications

Except for female sex, all the factors that predict high SR health are potentially modifiable. This includes the level of multimorbidity, Life Space Index score, and functional and psychological resilience. While the level of multimorbidity itself may not be modifiable, aspects of care, such as improved access to treatment, management of symptom or disease burden, and prevention of secondary disease can be achieved. This includes interventions and research that aim to address the social determinants of health [44], programs that tackle common risk factors such as alcohol or tobacco use, physical inactivity, and poor mental health [45], and approaches to enhance self-management capacity [46]. Additionally, Life Space Index as well as functional and psychological resilience are potentially modifiable. For example, the Life Space Index, a measure of community mobility, is related to modifiable factors such as social support and walking speed [47]. Previous research has demonstrated links between social support, walking speed, and important health outcomes, including known associations with walking speed and risk for falls and hospitalization [48, 49]. Similarly, functional resilience, captured as a composite score of physical and functional measures (including walking speed), and psychological resilience, comprised of depression, distress, and life satisfaction scales, are all factors that can be targeted and modified [50–52]. Building on the well-documented links between higher self-reported health and positive health outcomes for older adults [10], identification of these five key drivers has the potential to inform the development of clinical interventions that target these modifiable factors.

### Strengths and limitations

A key strength of this research involves the use of a large, population-based sample. This provided an opportunity to closely examine the relationship between sociodemographic, health-related, and resilience factors. However, the use of this dataset also contributed to some notable limitations that should be considered when interpreting findings. First, this research was a cross-sectional analysis of baseline data from the CLSA. As such, results cannot be interpreted as causal, nor can temporality of the factors or directionality of associations be captured. Second, while CLSA datasets are large and aim to be representative, there are limitations regarding certain demographic factors. For example, representation of race, for example, or the exclusion of certain population groups (e.g., veterans, individuals living in Canadian territories). This limits the application of these findings to broader populations as the sample for these analyses was predominantly white, English-speaking, urban-dwelling, and middle-income. Third, while level of multimorbidity is a widely used approach to measuring disease burden, the way that chronic conditions are captured in CLSA means that an individual may have a diagnosis of a specific condition (e.g., arthritis), however, that condition may not be causing any challenge or discomfort while for another person, that same condition may be very challenging or burdensome. By using a level of multimorbidity, as opposed to using a disease burden scale (e.g., Disability Adjusted Life Years [53]), there are limitations on the application of these findings. This is particularly true for those conditions which may relapse, remit, or carry a significant burden of illness such as stroke or chronic obstructive pulmonary disease. Fourth, there are limitations associated with the design of the CLSA. This includes limitations related to how the data is collected, as well as the duration of the study. It is likely that those who are willing to participate in a study lasting up to 20 years, particularly such a comprehensive study, may be different than those in the general population. As well, these analyses dominantly drew from the comprehensive dataset, meaning that individuals living in more rural communities outside of the data collection catchment areas, would not be included. Lastly, due to the large sample size, findings should be interpreted with emphasis on the effect of the relationship (e.g., Cohen’s classification criteria) and the general weakness of the models generated instead of solely the statistical significance reported.

## Conclusion

Self-reported health is one of the most commonly used outcome measures in epidemiology, health research, and clinical practice [54]. Findings from this study have highlighted that while many factors are associated with self-reported health, these factors do not seem to influence the relationship between multimorbidity and self-reported health. Findings have additionally identified the factors that predict high self-reported health are the same for the general population of older adults and a subset of this population with high multimorbidity. Further, this study has identified that of these five key factors, four of them are potentially modifiable including the level of multimorbidity, the Life Space Index score, and the functional and psychological resilience scores. Findings from this work have generated several additional research opportunities. This a need to leverage longitudinal studies using data from the CLSA to explore causal relationships (e.g., further examination of the temporality of factors), to repeat these analyses in differing populations (e.g., more diverse sample, a sample that includes more rural and remote participants), as well as to compare these findings to those who have the opposite of the well-being paradox (i.e., those with few or no chronic conditions and lower self-reported health). In addition, future qualitative research is warranted to explore how these key factors predict high self-reported health among community-dwelling older adults. Moving beyond an exploratory understanding of self-reported health and the well-being paradox, our findings have advanced understanding of the factors that predict high self-reported health among community-dwelling older adults.

## Supplementary Information

**Additional file 1.** Interaction effect figures.

## Data Availability

The data that support the findings of this study are available through the Canadian Longitudinal Study on Aging (CLSA) (www.clsa-elcv.ca) for researchers who meet the criteria for access to de-identified data.

## Abbreviations

ADL: Activities of daily living
ANOVA: Analysis of variance
CES-D-10: Centre for Epidemiologic Studies Depression Scale 10-item
CI: Confidence interval (95%, two-sided alpha)
CLSA: Canadian Longitudinal Study on Aging
IADL: Instrumental activities of daily living
MOS: Medical Outcomes Study
OARs: Older Americans Resources and Services
OR: Odds Ratio
SD: Standard Deviation
