# Supplementary material for: Self-reported health and the well-being paradox among community-dwelling older adults: a cross-sectional study using baseline data from the Canadian Longitudinal Study on Aging (CLSA)
Source: BMC Geriatr. 2022 Feb 10;22:112. doi: 10.1186/s12877-022-02807-z (PMC8832840; doi:10.1186/s12877-022-02807-z)
Supplement: Supplementary file 1 — Additional file 1. Interaction effect figures. [file 12877_2022_2807_MOESM1_ESM.docx]

**Supplementary File 1**


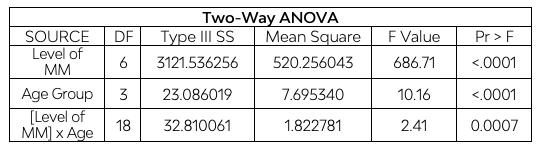

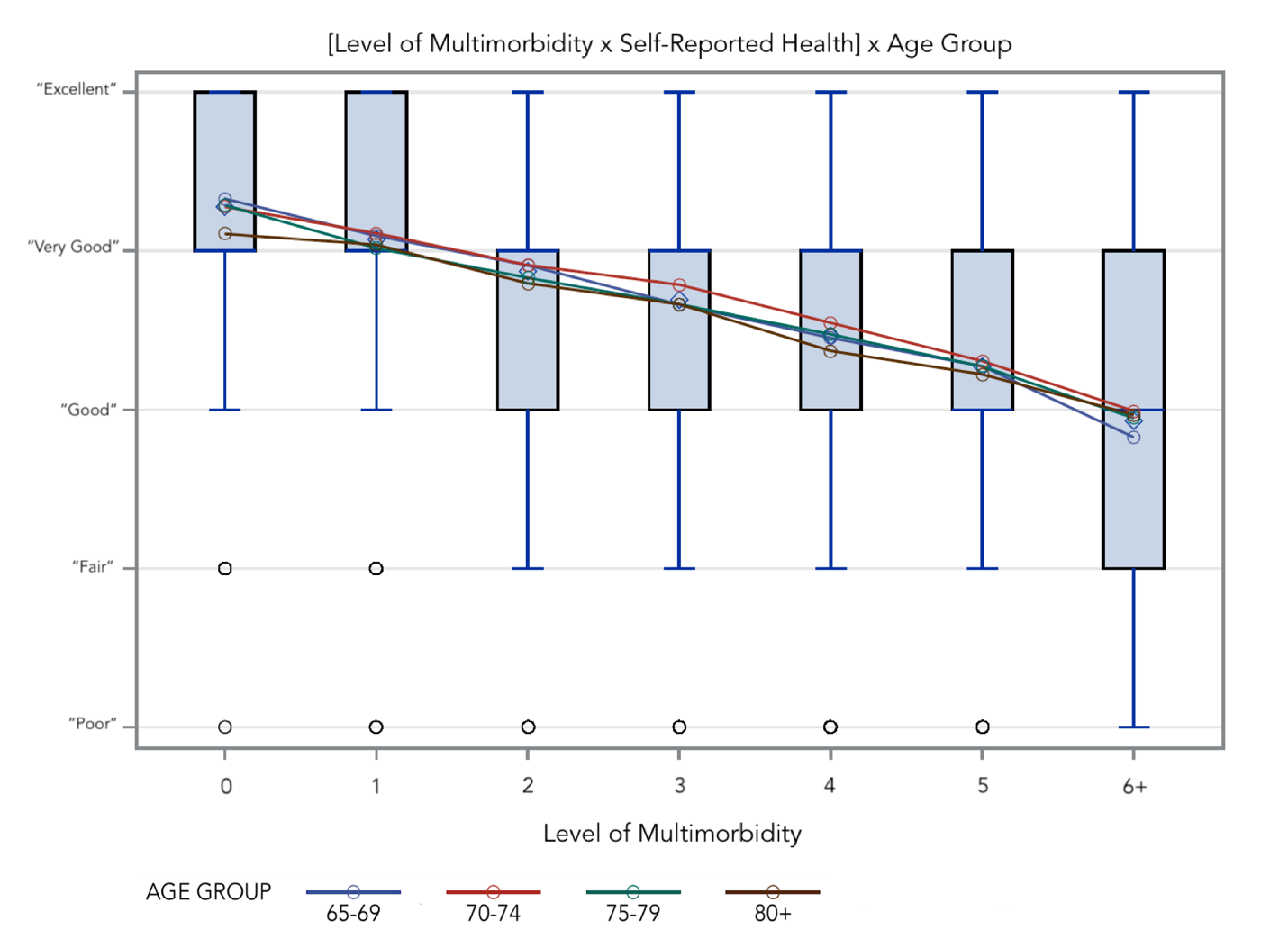
 *Interaction effect between self-reported health and the level of multimorbidity by age group.*


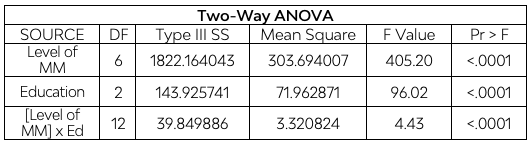
*
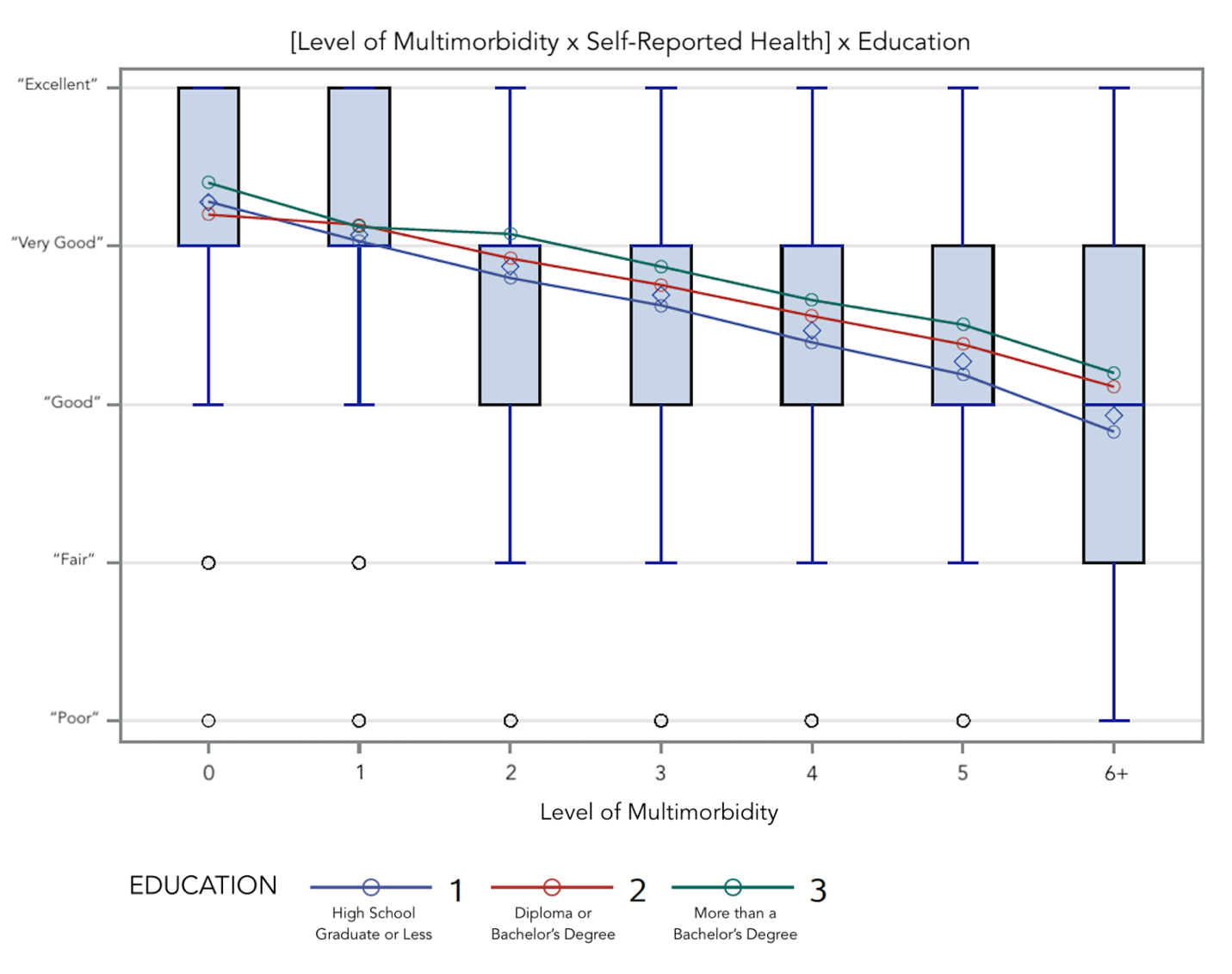
*

*Interaction effect between self-reported health and the level of multimorbidity by education.*

*
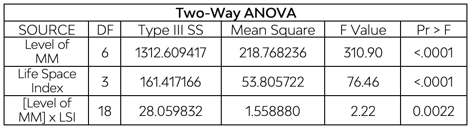

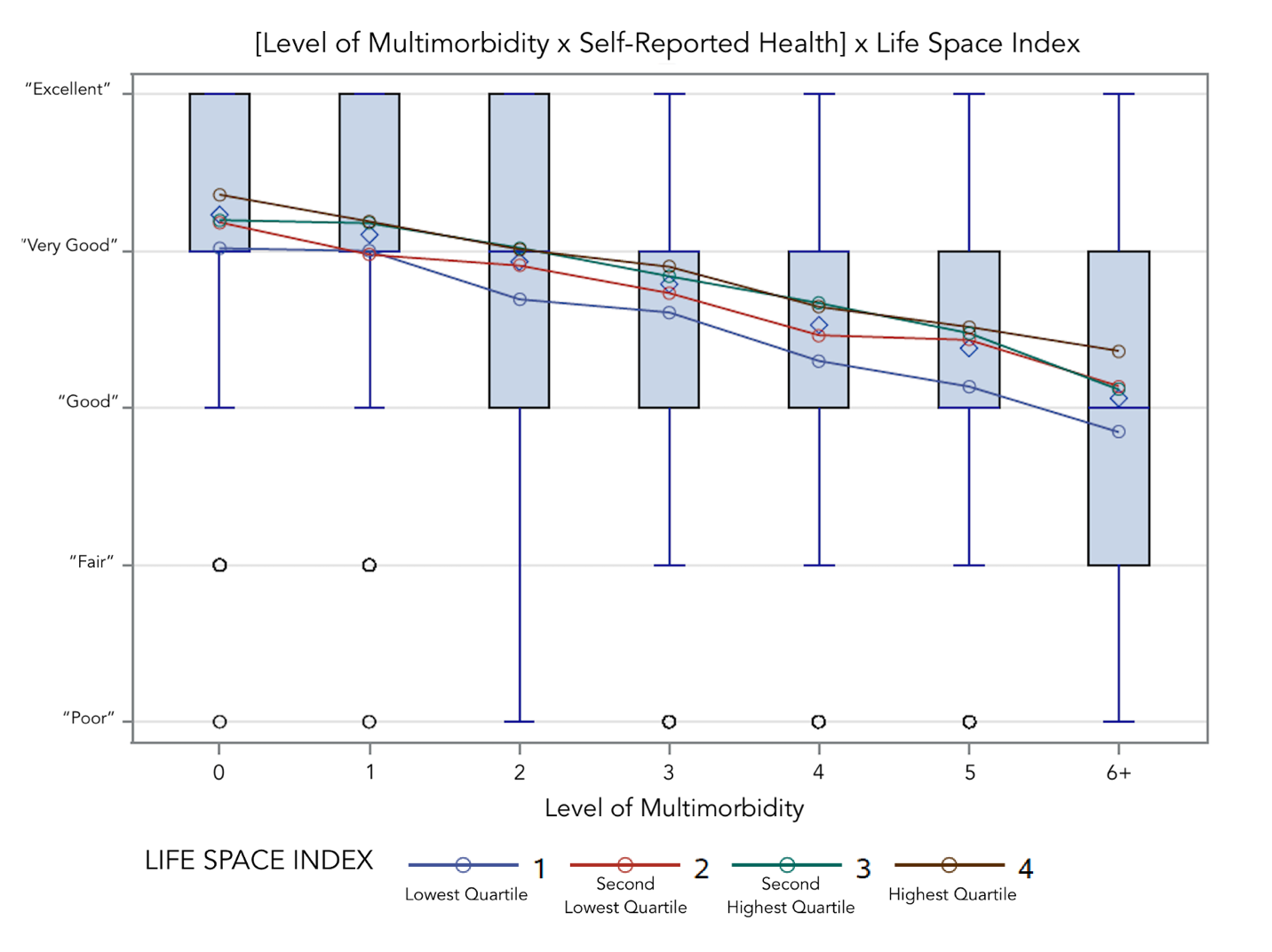
*

*Interaction effect between self-reported health and the level of multimorbidity by life space index quartiles (where the highest quartile represents a higher score).*
